# Phosphorylation of importin-α1 by CDK1–cyclin B1 controls mitotic spindle assembly

**DOI:** 10.1242/jcs.232314

**Published:** 2019-09-15

**Authors:** Li Guo, Khamsah Suryati Mohd, He Ren, Guangwei Xin, Qing Jiang, Paul R. Clarke, Chuanmao Zhang

**Affiliations:** 1 The MOE Key Laboratory of Cell Proliferation and Differentiation, College of Life Sciences, Peking University, Beijing 100871, China; 2 School of Medicine, Jacqui Wood Cancer Centre, Ninewells Hospital and Medical School, University of Dundee, Dundee DD1 9SY, UK; 3 The University of Queensland Diamantina Institute, The University of Queensland, Woolloongabba, Brisbane, QLD 4102, Australia

**Keywords:** Mitosis, Cell cycle, Spindle assembly, Cyclin-dependent kinase, Importin

## Abstract

Importin-α serves as an adaptor linking importin-β to proteins carrying a nuclear localization sequence (NLS). During interphase, this interaction enables nuclear protein import, while in mitosis it regulates spindle assembly factors (SAFs) and controls microtubule nucleation, stabilization and spindle function. Here, we show that human importin-α1 is regulated during the cell cycle and is phosphorylated at two sites (threonine 9 and serine 62) during mitosis by the major mitotic protein kinase CDK1–cyclin B. Mutational analysis indicates that the mitotic phosphorylation of importin-α1 inhibits its binding to importin-β and promotes the release of TPX2 and KIFC1, which are then targeted like importin-β to the spindle. Loss of importin-α1 or expression of a non-phosphorylated mutant of importin-α1 results in the formation of shortened spindles with reduced microtubule density and induces a prolonged metaphase, whereas phosphorylation-mimicking mutants are functional in mitosis. We propose that phosphorylation of importin-α1 is a general mechanism for the spatial and temporal control of mitotic spindle assembly by CDK1–cyclin B1 that acts through the release of SAFs such as TPX2 and KIFC1 from inhibitory complexes that restrict spindle assembly.

## Abstract (precis)

**Summary:** Phosphorylation of importin-α1 by cyclin-dependent kinase 1 provides spatial and temporal control of mitotic spindle assembly through the release of spindle-assembly factors from inhibitory complexes during mitosis.

## INTRODUCTION

The mitotic spindle, composed mainly of microtubules (MTs) and MT-associated proteins (MAPs), functions to distribute the duplicated genome to daughter cells during cell division (Petry, 2016). Spindle assembly can be driven through a centrosome-nucleated pathway in which MTs with the property of dynamic instability interact with chromosomes through a ‘search-and-capture’ process and become stabilized by interaction with kinetochores (Heald and Khodjakov, 2015). A chromatin-mediated pathway promotes spindle assembly through localized generation of Ran-GTP by the guanine-nucleotide exchange factor RCC1 (Bischoff and Ponstingl, 1991), which is localized to chromosomes during mitosis (Moore et al., 2002). Hydrolysis of GTP by Ran, stimulated by cytoplasmic RanGAP1 (Bischoff et al., 1994), further restricts Ran-GTP to the vicinity of chromosomes, forming a gradient signal that promotes MT nucleation, stabilization and spindle assembly around chromosomes (Caudron et al., 2005; Kaláb et al., 2006, 2002) through release of spindle-assembly factors (SAFs) from inhibitory complexes with importins (Bamba et al., 2002; Gruss et al., 2001; Nachury et al., 2001; Wiese et al., 2001).

Importin-α contains an importin-β binding (IBB) domain in its N-terminus and two nuclear localization sequence (NLS)-binding sites (major and minor) in its C-terminus. It serves as a linker between importin-β and NLS-containing cargo to form a trimeric complex that is transported into the nucleus (Cingolani et al., 1999; Conti and Kuriyan, 2000; Conti et al., 1998; Giesecke and Stewart, 2010; Kobe, 1999). Importin-α has also been shown to play a role in the control of mitotic spindle assembly in *Xenopus* egg extracts (Gruss et al., 2001; Schatz et al., 2003) and it is essential for this process in *C. elegans* (Askjaer et al., 2002). During mitosis, importin-α binds NLS motifs in SAFs such as TPX2, TACC3, NuMA (also known as NUMA1) and KIFC1, forming a trimeric complex with importin-β that restricts the function of the SAF. Once importin-β encounters Ran-GTP around chromosomes, it switches conformation, dissolving the trimer and releasing the SAF to function in mitotic spindle assembly (reviewed in Clarke and Zhang, 2008). Seven importin-α isoforms have been identified thus far in humans grouped into three subfamilies, α1, α2 and α3. While other importin-α isoforms recognize specific non-classical cargoes, the most conserved isoform, importin-α1 (encoded by *KPNA2*) recognizes classical NLSs for nuclear import and is likely to be the major regulator of SAFs in mitosis (Pumroy and Cingolani, 2015).

Spindle assembly also requires the activation of mitotic protein kinases, notably CDK1–cyclin B1, which phosphorylates key proteins involved in nuclear envelope breakdown, chromatin condensation and spindle formation (Nigg, 2001). CDK1–cyclin B1 also controls the Ran system through modification of its regulators. Phosphorylation of mammalian RCC1 by CDK1–cyclin B1 inhibits the binding of importin-α3 to the NLS motif in C-terminal tail of RCC1, enabling RCC1 to interact with chromatin and thereby promoting the generation of Ran-GTP on mitotic chromosomes (Hutchins et al., 2004; Li and Zheng, 2004). RanGAP1 is also phosphorylated by CDK1–cyclin B1, enabling interaction with RanBP2 and the SUMO-conjugating enzyme Ubc9 in mitosis (Swaminathan et al., 2004). Furthermore, mitotic spindle assembly is promoted by the recruitment of RanGAP1 and RanBP2 to the spindle through phosphorylation of the nuclear transport factor CRM1 by CDK1–cyclin B1 (Wu et al., 2013). Phosphorylation of specific SAFs, such as TPX2, by mitotic kinases can regulate their function in mitotic spindle assembly (Bian et al., 2010; Fu et al., 2015, 2010) and phosphorylated SAFs can, in turn, influence the enzymatic activities, localization and functions of mitotic kinases (Kufer et al., 2002). However, it has been unclear whether there is a general mechanism for the control of SAFs by mitotic kinases.

In this work, we have found that importin-α1 is regulated during the cell cycle and is phosphorylated during mitosis by CDK1–cyclin B1 at two sites. These modifications negatively regulate its binding to importin-β and NLS-containing SAFs, thereby releasing the active SAF to promote mitotic spindle assembly. These results identify a mechanism for the induction of mitotic spindle assembly by CDK1–cyclin B1 through the control of inhibitory complexes with importin-α1.

## RESULTS

### Importin-α1 is highly expressed in mitosis and is required for proper spindle assembly

**Fig. 1.**
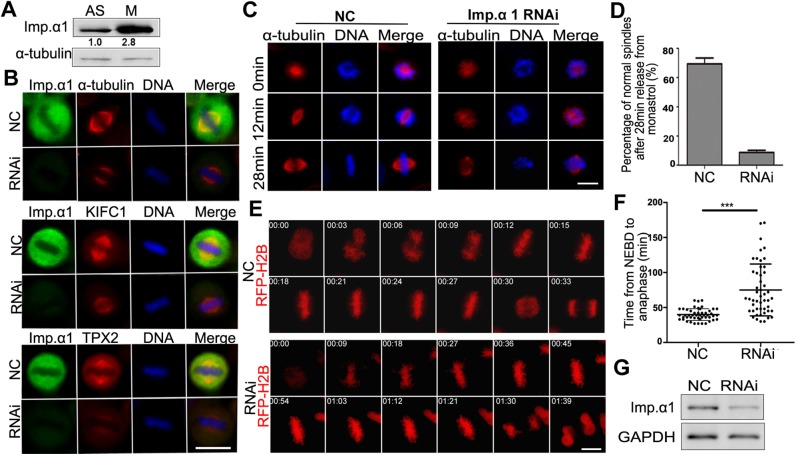
**Human importin-α1 is required for mitotic spindle assembly.** (A) The expression level of importin-α1 is increased in mitosis. Cell extracts from asynchronous and nocodazole-arrested mitotic HeLa cells were prepared, subjected to SDS-PAGE and analyzed by immunoblotting with anti-importin-α1 and α-tubulin antibodies. The numbers below the bands refer to the relative gray value intensity. (B) HeLa cells were transfected with negative control (NC) siRNA or importin-α1 siRNA at 100 nM for 72 h. Cells were fixed and immunostained using antibodies against importin-α1, α-tubulin, KIFC1 and TPX2. DNA (blue) was stained using DAPI. Typical images are shown. (C) Monastrol-released MT regrowth was affected under importin-α1 knockdown. HeLa cells were treated with NC siRNA or importin-α1 siRNA for 72 h and were released into warm medium for 12 min and 28 min from monastrol-arrested mitosis and then were fixed and immunostained with anti-α-tubulin antibody. (D) Percentage of normal spindle formation 28 min after release from monastrol. Experiments were repeated three times, and at least 100 cells were measured for each group. Results are mean±s.d. (E) Importin-α knockdown results in a prolonged metaphase arrest in HeLa cells. HeLa cells were transfected with siRNA and RFP–H2B (as a chromatin marker, red) for 72 h and were subjected to automated time-lapse live-cell imaging. The onset of NEBD is marked as 0 min. (F) The average time from NEBD to sister chromosome separation of cells in E was calculated and statistically analyzed. Results are mean±s.d., *n*=50 cells per group. ***P<0.001 (Student's *t*-test). (G) Analysis of the efficiency of siRNA knockdown by immunoblotting using anti importin-α1 and GAPDH antibodies. Scale bars: 10 µm.

To investigate the functions of importin-α1 during the cell cycle, we first analyzed the expression levels of importin-α1 in human HeLa cells and we found that the protein is expressed 2- to 3-fold more highly in mitosis than in interphase (Fig. 1A). When cells were synchronized at different stages of the cell cycle we found that, while importin-β was maintained throughout the cell cycle, the relatively high level of importin-α1 in early G1 was reduced in S and G2 phases and then increased in mitosis (Fig. S1A). This result was also confirmed in experiments where cells where subjected to a G1/S or M phase block and release, followed by western blotting analysis, which showed that the levels of importin-α1 correlated with those of the mitotic marker phosphorylated histone H3 (Fig. S1B,C).

As expected, endogenous importin-α1 and GFP-tagged importin-α1 were expressed mainly in the cytoplasm during interphase, with some localization to the nuclear envelope and in the nucleus. In mitosis, importin-α1 was dispersed throughout the cytoplasm, with a weak concentration on the mitotic spindle, and was excluded from the chromosomes (Fig. 1B; Fig. S2). The specificity of immunofluorescence was confirmed by knockdown of endogenous importin-α1 after exposure to siRNA. In importin-α1-knockdown cells, we found that mitotic spindles were significantly shortened, and the mean intensity of the spindle MTs was reduced (Fig. 1B; see also Fig. S6). Furthermore, we observed that two SAFs, the kinesin 14 family motor protein KIFC1, also known as HSET (Mountain et al., 1999; Zhu et al., 2005), and the MT-associated protein TPX2 (Garrett et al., 2002; Gruss et al., 2001, 2002; Kufer et al., 2002; Schatz et al., 2003; Trieselmann et al., 2003; Wittmann et al., 2000), were only weakly localized to the shortened spindles of the importin-α1-knockdown cells (Fig. 1B). We also treated cells with monastrol, an inhibitor of the kinesin Eg5 (also known as KIF11), to arrest the cells in mitosis with monopolar spindles (Mayer et al., 1999) and then released them to allow their bipolar spindle assembly. The results showed that the bipolar spindle assembly in importin-α1-knockdown cells was very slow and the assembled bipolar spindle was obviously abnormal (Fig. 1C,D,G), indicating that importin-α1 is required for spindle assembly. Through live-cell imaging, we observed that importin-α1 knockdown resulted in a significantly prolonged metaphase arrest (Fig. 1E–G), suggesting that proper chromosome attachment and satisfaction of the spindle assembly checkpoint (Musacchio, 2015) requires importin-α1. These results suggest that the high expression level of importin-α1 during mitosis enables proper bipolar spindle assembly.

### Importin-α1 is phosphorylated at T9 and S62 in mitosis by CDK1–cyclin B1

**Fig. 2.**
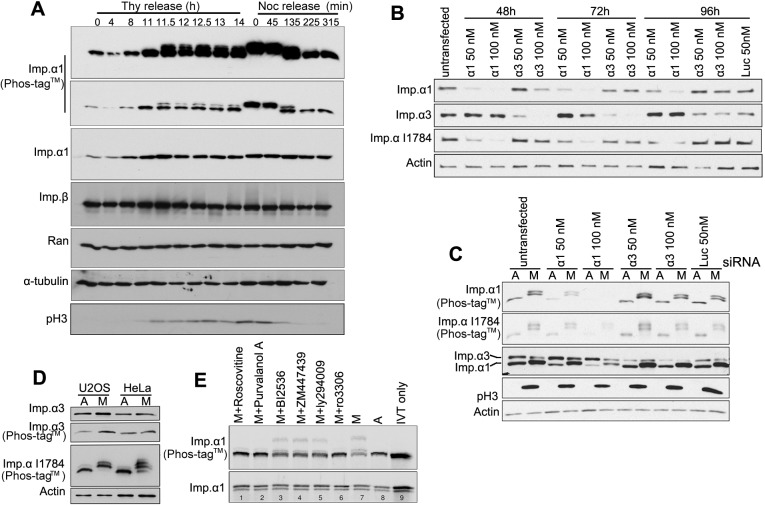
**Phosphorylation of importin-α1 during the cell cycle.** (A) HeLa cells were arrested at G1/S via a double-thymidine blockade, followed by release at different time points, or were arrested at mitosis via a double-thymidine blockade, followed by release into nocodazole blockade and release at different time points. Cells were collected and subjected to Phos-tag™ SDS-PAGE and standard SDS-PAGE and immunoblotting. Histone H3 phosphorylated at S10 (pH3) was detected to indicate mitosis. α-Tubulin serves as a loading control. (B) Importin-α1 is the most abundant isoform recognized by anti-importin-α antibody I1784. HeLa cells were transfected with importin-α1, importin α3 and luciferase siRNA at the indicated concentration for 48–96 h. Western blotting using the mentioned antibodies revealed the knockdown efficiency. (C) Importin-α1 is the main isoform phosphorylated in mitosis. HeLa cells were transfected with importin-α1, importin α3 and luciferase siRNA at the indicated concentration for 96 h. Asynchronous and mitotic cell lysates were subjected to standard and Phos-tag™ SDS-PAGE, followed by western blotting using the indicated antibodies. (D) Importin-α3 is not phosphorylated in mitosis. Asynchronous and mitotic cell lysates of U2OS and HeLa cells were subjected to standard and Phos-tag™ SDS-PAGE, followed by western blotting using anti-importin-α3 (Abcam, ab6039) and anti-importin-α I1784 antibodies. Actin served as a loading control. (E) IVT importin-α1 phosphorylation is blocked by CDK1 inhibitors. IVT-imp-α1 was incubated in mitotic cell extracts with the addition of a range of kinase inhibitors for 30 min at 30°C. Reactions were stopped by the addition of 2× SDS sample buffer, boiled for 3 min and spun for 5 min. The resultant supernatants were subjected to standard and Phos-tag™ SDS-PAGE. Autoradiography of Phos-tag™ gels showed that the CDK1 inhibitors roscovitine, purvalanol A and Ro3306 blocked IVT-imp-α1 phosphorylation. A, asynchronous; M, mitotic; IVT only, recombinant protein alone without treatment.

Next, we investigated the possible regulation of importin-α1 during the cell cycle. Cells were blocked at G1/S (with thymidine) or mitosis (with nocodazole) followed by release into the cell cycle. Western blot analysis of SDS-PAGE gels incorporating Phos-tag™ showed that importin-α1 was phosphorylated when cells entered mitosis and that dephosphorylation occurred when the cells exited mitosis (Fig. 2A). By using specific antibodies against importin-α1 and importin-α3 in combination with an antibody against importin-α (I1784; Sigma-Aldrich), which has been reported to recognize importin-α1, importin-α3, importin-α5 and importin-α7, we found that the knockdown of the most abundant importin-α1, but not importin-α3 (encoded by *KPNA4*), greatly reduced the upshifted bands of mitotic importin-α recognized by I1784 in Phos-tag™ blotting (Fig. 2B,C), indicating that importin-α1 is the main isoform that is phosphorylated in mitosis. Moreover, no upshifted bands were seen in the mitotic lysates detected by anti-importin-α3 antibody on Phos-tag™ gel blots (Fig. 2D), indicating that the less-abundant isoform importin-α3 is not phosphorylated in mitosis.

To verify whether importin-α1 phosphorylation is functionally significant, we set out to identify the kinase that phosphorylates importin-α1 in mitosis. *In vitro* translated (IVT) importin-α1 was incubated with asynchronous or mitotic HeLa extracts with the addition of various kinase inhibitors. The results showed that the pan-CDK inhibitors roscovitine and purvalanol A, as well as the CDK1 inhibitor RO3306, effectively blocked mitotic phosphorylation of IVT importin-α1, while the PLK1 inhibitor BI2536, the Aurora kinase inhibitor ZM447439 and the phosphoinositide 3-kinase (PI3K) inhibitor LY294009 did not affect the mitotic phosphorylation of IVT importin-α1 (Fig. 2E). These results indicated that CDK1 in complex with its primary mitotic activating subunit, cyclin B1 (Nigg, 2001), is required for the phosphorylation of importin-α1 in mitosis.

**Fig. 3.**
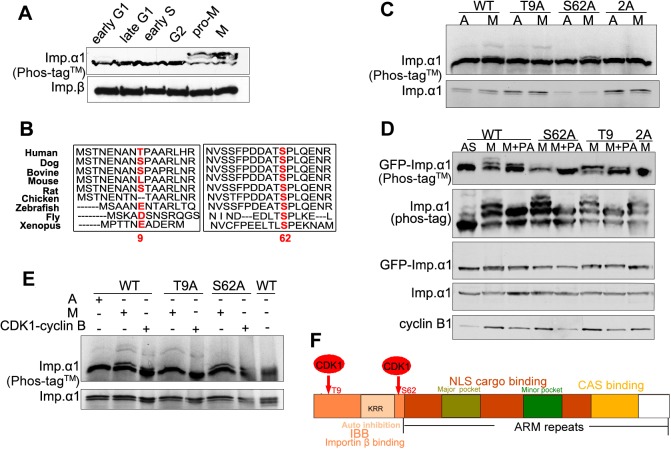
**Importin-α1 is phosphorylated at T9 and S62 by CDK1–cyclin B1 in mitosis.** (A) Importin-α1 is phosphorylated in mitosis at at least two sites. Early S and G2 phase cell extracts were prepared from synchronous HeLa cells at 1 h and 8 h after release from double-thymidine blockade; M phase, early G1 and late G1 cell extracts were prepared from synchronized HeLa cells at 0 h, 4 h and 9 h after release from nocodazole arrest. Cell extracts were subjected to Phos-tag™ SDS-PAGE and were analyzed by immunoblotting with antibodies as indicated. (B) Sequence alignment of importin-α1 among different species. Alignment of the N-terminal region of importin-α1 among different species performed using the CLUSTAL OMEGA multiple alignment tool on the EMBL-EBI website (http://www.ebi.ac.uk/Tools/msa/clustalo/). T9 and S62 are highlighted in red. UniProt identifiers: IMA1_HUMAN, E2R6L9_CANFA, Q3SYV6_BOVIN, IMA1_MOUSE, Q9Z0N9_RAT, F1NJS6_CHICK, Q6DI01_DANR, IMA_DROME, IMA1_XENLA. (C) HeLa cells were transiently transfected with GFP–importin-α1 WT, GFP–importin-α1 S62A, GFP–importin-α1 T9A or GFP-importin-α1 2A. After 17 h of treatment with 100 ng/ml of nocodazole, cells were treated with the CDK inhibitor purvalanol A (+PA) for 20 min. Asynchronous (labeled A or AS) and nocodazole-arrested mitotic cell lysates (labeled M) were collected as controls. Cell extracts were subjected to Phos-tag™ and standard SDS-PAGE and immunoblotting with the indicated antibodies. (D) T9 and S62 double mutation abolished the mitotic phosphorylation of importin-α1. IVT importin-α1 WT, alanine and double-alanine mutants were incubated in mitotic cell extracts for 30 min at 30°C. The reactions were stopped by the addition of 2× SDS sample buffer, boiled for 3 min and centrifuged for 5 min. The resultant supernatants were subjected to standard and Phos-tag™ SDS-PAGE. Autoradiograph of the Phos-tag™ gel showed that double-alanine mutation of T9 and S62 abolished the mitotic phosphorylation of IVT importin-α1. (E) Phosphorylation of IVT importin-α1 by purified CDK1–cyclin B1. IVT importin-α1 WT, T9A and S62A were incubated with asynchronous, mitotic cell extracts or purified recombinant CDK1–cyclin B1 protein for 30 min at 30°C. Reactions were stopped by the addition of 2× SDS sample buffer, boiled for 3 min and centrifuged for 5 min. The resultant supernatants were subjected to standard and Phos-tag™ SDS-PAGE. Autoradiography of a Phos-tag™ gel showed that CDK1–cyclin B1 could phosphorylate IVT importin-α1 in the same manner as the cell extracts. Point mutations at T9 and S62 also blocked the phosphorylation by CDK1–cyclin B1. CDK1–cyclin B1 storage buffer was used as the control to eliminate a possible non-specific effect on phosphorylation. (F) A schematic diagram showing the functional domains and CDK1 phosphorylation sites of importin-α1.

Two prominent retarded forms of importin-α1 were observed Phos-tag™ gel blots of mitotic cells (Fig. 3A), indicating at least two mitotic phosphorylation sites. Based on bioinformatical analysis, we chose to focus on threonine 9 (T9) and serine 62 (S62), given that the sequence flanking the importin-α1 T9 and S62 conform to the preferred consensus substrate motif (pS/T-P) of CDK1–cyclin B1 (Johnson, 2011). Whereas T9 is only present in human importin-α1, S62 is highly conserved among importin-α1 homologs in eukaryotes (Fig. 3B). Both sites have been identified as phosphorylated residues in multiple global phosphoproteomic analyses of cells arrested in mitosis (Hornbeck et al., 2015). By expressing GFP–importin-α1 wild-type (WT) and mutants S62A, T9A and the double mutant S62A/T9A (2A) in HeLa cells or by incubating IVT importin-α1 (WT, S62A, T9A and 2A) with HeLa extracts, we found that the T9A and S62A mutations each abolished one of two upshifted bands of GFP–importin-α1 or IVT importin-α1 on Phos-tag™ SDS-PAGE gels, while the double mutant 2A abrogated all the mitotic upshifted bands (Fig. 3C). Treatment with the CDK inhibitor purvalanol A (PA) also further abolished the remaining slower migrating band for T9A and S62A (Fig. 3D), indicating that importin-α1 is modified by a CDK at both sites. Indeed, IVT importin-α1 can be directly phosphorylated by purified CDK1–cyclin B1, and this phosphorylation was abolished for the 2A mutation and the phosphorylation of T9A and S62A single mutants was also affected (Fig. 3E). These results indicate that CDK1–cyclin B1 is responsible for the phosphorylation of importin-α1 at T9 and S62 during mitosis. These sites lie in the N-terminal region of importin-α1 which contains an importin-β binding domain (IBB) (Fig. 3F).

### Phosphorylation of importin-α1 attenuates the interaction with importin-β and NLS-containing SAFs

**Fig. 4.**
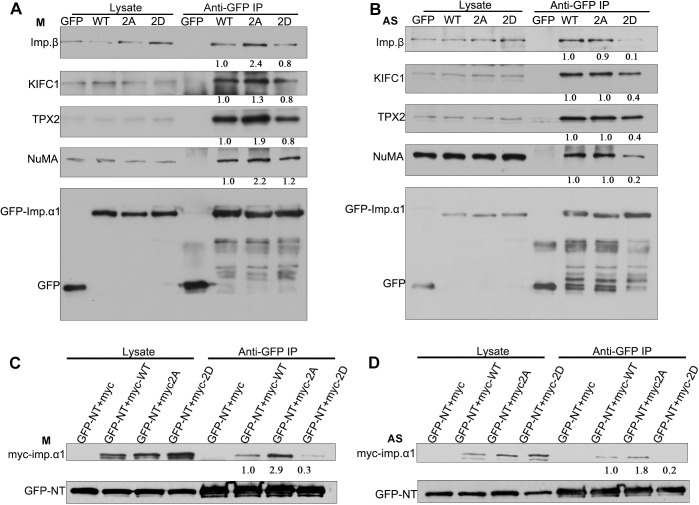
**Phosphorylation at T9 and S62 attenuates the interaction of importin-α1 with importin-β and NLS-containing SAFs in mitosis.** (A) HeLa cells were transiently transfected with GFP, GFP–importin-α1 WT, GFP-importin-α1 2A or GFP–importin-α1 2D and were arrested in mitosis (labeled M) with 100 ng/ml of nocodazole for 17 h. GFP–importin-α1 was immunoprecipitated (IP) from mitotic cells using anti-GFP antibody, followed by immunoblotting for importin-β, KIFC1, TPX2, NuMA and GFP. Cell lysates used for the precipitations are shown in the left panels. (B) HeLa cells were transiently transfected with either GFP, GFP–importin-α1 WT, GFP–importin-α1 2A or GFP–importin-α1 2D and were collected as asynchronous (labeled AS) cells. GFP–importin-α1 was immunoprecipitated from asynchronous cells using the anti-GFP antibody, followed by immunoblotting for importin-β, KIFC1, TPX2, NuMA and GFP. Cell lysates used for the precipitations are shown in the left panels. (C) T9 and S62 phosphorylation may function in an NLS-dependent manner in mitosis. HeLa cells were co-transfected with the NLS-containing fragment GFP–NT and Myc-tagged importin-α1 or mutants [Myc-importin-α1 WT (myc-WT), the non-phosphorylation-mimicking double-mutant Myc-importin-α1 2A (myc-2A) and phosphorylation-mimicking double-mutant Myc-importin-α1 2D (myc-2D)] and then were arrested in mitosis with 100 ng/ml of nocodazole for 17 h. GFP–NT was immunoprecipitated from mitotic cells using an anti-GFP antibody, followed by immunoblotting for Myc and GFP. (D) HeLa cells were co-transfected with the NLS-containing fragment GFP–NT and Myc-tagged importin-α1 or mutants as in C, and then were collected as asynchronous cells. GFP–NT was immunoprecipitated from asynchronous cells using an anti-GFP antibody, followed by immunoblotting for Myc and GFP. The numbers under the bands refer to the relative grey value intensity.

To test whether the phosphorylation of importin-α1 affects its interaction with importin-β and its NLS-containing partners, lysates of mitotic HeLa cells transiently expressing GFP–importin-α1, GFP–importin-α1 2A and GFP–importin-α1 2D were immunoprecipitated using an anti-GFP antibody, followed by western blotting using anti-importin-β, -KIFC1, -TPX2, -NuMA and -GFP antibodies. We found that the non-phosphorylated double-mutant GFP–importin-α1 2A had a stronger binding affinity with importin-β, KIFC1, TPX2 and NuMA, while GFP–importin-α1 and the phosphorylation-mimicking double-mutant GFP–importin-α1 2D had reduced interactions with importin-β and those three SAFs in mitosis (Fig. 4A). The single mutants GFP–importin-α1 T9A and GFP–importin-α1 S62A also showed the same result, binding more SAFs than the wild-type GFP-importin-α1, as above (Fig. S3A–C). These results suggest that the phosphorylation of importin-α1 at T9 and S62 by CDK1–cyclin B1 in mitosis reduces the binding of importin-α1 to importin-β and its substrates. Consistent with these results, in asynchronous HeLa cells, GFP–importin-α1 2D had a similarly reduced interaction with importin-β and its substrates, while there was no significant difference among the WT, T9A, S62A and 2A mutants (Fig. 4B; Fig. S3A–C).

To test whether the phosphorylation of importin-α1 at T9 and S62 affects its interaction with NLS-containing substrates, lysates of mitotic HeLa cells co-expressing GFP–NT, a typical NLS-containing sequence from NuMA (Lu et al., 2012), were incubated with Myc–importin-α1 WT, Myc–importin-α1 2A or Myc–importin-α1 2D were immunoprecipitated using anti-GFP antibody, followed by western blotting analysis. The results showed that Myc–importin-α1 2A can bind this typical NLS-containing sequence more strongly than importin-α1 WT and 2D (Fig. 4C). It is also the case that the single-point mutants Myc-importin-α1 T9A and S62A had a stronger interaction with NLS (Fig. S3D). These results indicate that phosphorylation at T9 and S62 by CDK1–cyclin B1 can attenuate the interaction between importin-α1 and its substrates in an NLS-dependent manner in mitosis. Consistent with these results, Myc–importin-α1 2D had similarly reduced interaction with importin-β and its substrates, while there was no significant difference among the WT, T9A, S62A and 2A mutants in asynchronous HeLa cells (Fig. 4D; Fig. S3E).

### Expression of importin-α1 non-phosphorylated mutants results in prolonged metaphase in HeLa cells

**Fig. 5.**
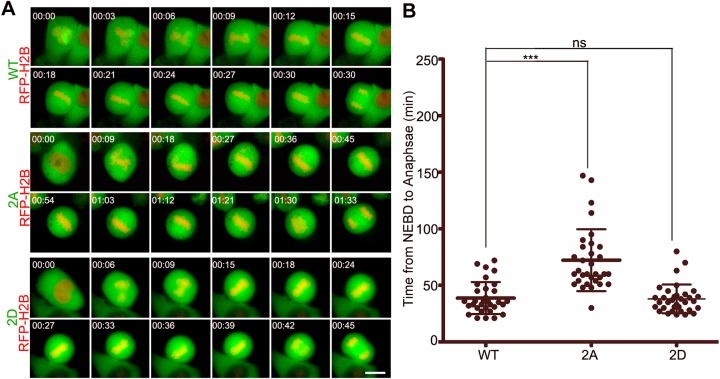
**Phosphorylation of importin-α1 at T9 and S62 is required for mitotic progression.** (A) HeLa cells were co-transfected with GFP–importin-α1 WT or mutants (2A and 2D) and RFP–H2B (as a chromatin marker) and then were subjected to automated time-lapse live-cell fluorescence imaging. The GFP signals indicate cells transfected with GFP importin-α1 or mutants. The red signals indicate H2B. The onset of NEBD is marked as 0 min. Scale bar: 10 µm. (B) Statistics showed that the average time from NEBD to anaphase was prolonged by non-phosphorylation-mimicking mutant GFP-importin-α1 2A. Results are mean±s.d., *n*=50 cells per group. ***P<0.001; ns, not significant (Student's *t*-test).

To test whether the phosphorylation of importin-α1 is important for mitotic spindle assembly, HeLa cells were transfected with GFP–importin-α1 plasmids for 24 h and then were prepared for live-cell imaging. The results showed that the cells expressing GFP–importin-α1 T9A, S62A and 2A had a significantly prolonged metaphase, while those expressing WT, the phosphorylation-mimicking double-mutant 2D (Fig. 5) or the single site phospho-mimicking mutants T9E or S62E (Fig. S4A–C) progressed to anaphase normally. However, once anaphase was initiated, cells exited mitosis on schedule regardless of whether they were expressing GFP-importin-α1 2A, T9E or S62E mutants (Fig. S4D). We also found that the nuclear localization of GFP–importin-α1 2D was reduced compared with that of the WT and non-phosphorylated mutant 2A in interphase, presumably because it disrupts the interaction of importin-α1 with nuclear transport complexes, whereas no alteration of the spindle localization of importin-α1 was observed in mitosis (Fig. S5). Taken together, these data demonstrate that the phosphorylation of importin-α1 at T9 and S62 by CDK1–cyclin B1 is specific and functional in mitosis but not in interphase.

### Phosphorylation of importin-α1 is required for mitotic spindle assembly

**Fig. 6.**
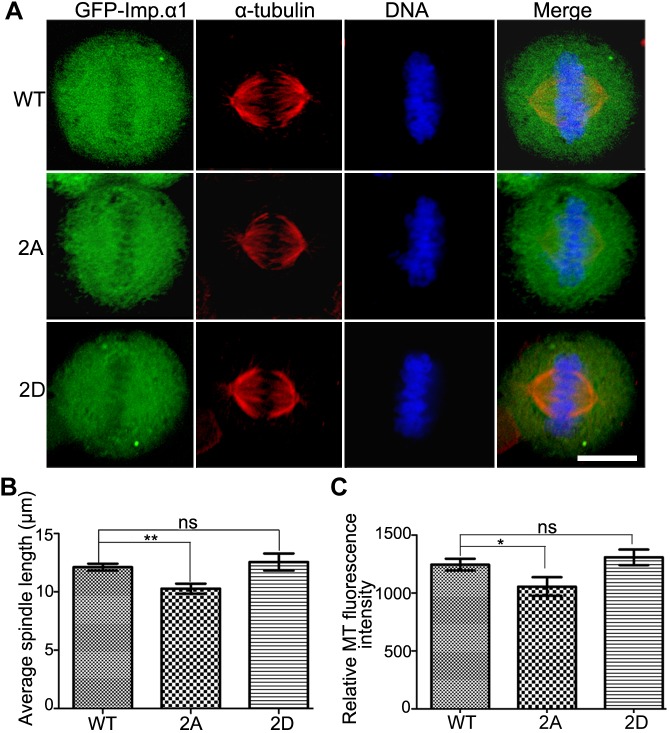
**Inhibition of importin-α1 phosphorylation at T9 and S62 prevents normal mitotic spindle assembly.** (A) HeLa cells were transfected with GFP–importin-α1 WT, GFP–importin-α1 2A and GFP-importin-α1 2D for 48 h and then were fixed with methanol and immunostained with anti-α-tubulin antibody. DNA (blue) was stained using DAPI. Typical images are shown. Scale bar: 10 µm. (B) The spindle length was measured with Volocity software (Perkin Elmer). Statistical quantifications were performed using GraphPad Prism5 software. (C) The MT intensity was measured by Volocity software (Perkin Elmer). Statistical quantifications were performed using GraphPad Prism5 software. For B and C, results are mean±s.d. Experiments were repeated three times, and at least 100 cells were measured for each group. *P<0.05; **P<0.01; ns, not significant (Student's *t*-test).

Upon expression of GFP–importin-α1 WT, 2A and 2D in cells, we found that the non-phosphorylated mutant 2A affected mitotic spindle assembly in cells with shortened spindle formation and weakened the MT intensity, while the WT and the phospho-mimicking double-mutant 2D mutant had no such effects (Fig. 6). These results suggest that the phosphorylation of importin-α1 plays a positive role in mitotic spindle assembly.

**Fig. 7.**
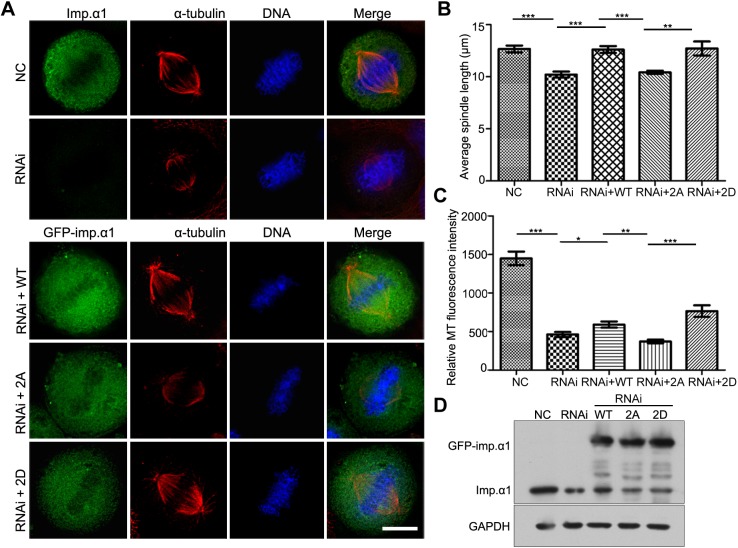
**Phosphorylation of importin-α1 at T9 and S62 is required for normal spindle assembly.** (A) HeLa cells were transfected with siRNA and siRNA-resistant plasmids (GFP-WT, GFP-2A and GFP-2D) to rescue the knockdown of importin-α1, followed by fixation with methanol and immunostaining with an anti-α-tubulin antibody. DNA was stained using DAPI. Scale bar: 10 µm. (B) The spindle length was measured by Volocity software (Perkin Elmer). Results are mean±s.d. Experiments were repeated three times, and at least 100 cells were measured for each group. (C) Analysis of relative MT fluorescence intensity as shown in A was performed using GraphPad Prism5 software. Results are mean±s.d. with at least 50 cells were measured for each group. Experiments were repeated three times. (D) Analysis of the efficiency of siRNA knockdown and rescue by immunoblotting. *P<0.05, **P<0.01, ***P<0.001 (Student's *t*-test).

Knowing that the knockdown of importin-α1 resulted in shortened spindles, reduced intensity of MTs, impaired localization of SAFs on spindle and prolonged metaphase arrest, we carried out knockdown-and-rescue experiments to further investigate the role of phosphorylation of importin-α1 in mitotic spindle assembly. HeLa cells were exposed to siRNA and siRNA-resistant GFP–importin-α1, GFP–importin-α1 2A and GFP–importin-α1 2D plasmids for 72 h. Through measuring the relative MT fluorescence intensity and the pole-to-pole length of the metaphase spindles, we found that the non-phosphorylated mutant 2A was completely unable to restore the shortened spindle caused by siRNA, while WT and 2D almost restored the spindle length to a normal level (Fig. 7A,B). Moreover, although the WT and 2D forms could slightly restore the reduced intensity of MTs caused by siRNA to the level of cells containing endogenous importin-α1, 2A could not restore the effect of siRNA (Fig. 7A,C). Thus, we conclude that CDK1-dependent phosphorylation of importin-α1 at T9 and S62 is indispensable in mitotic spindle assembly.

### Phosphorylation of importin-α1 is necessary for the localization of importin-β and SAFs to the mitotic spindle

**Fig. 8.**
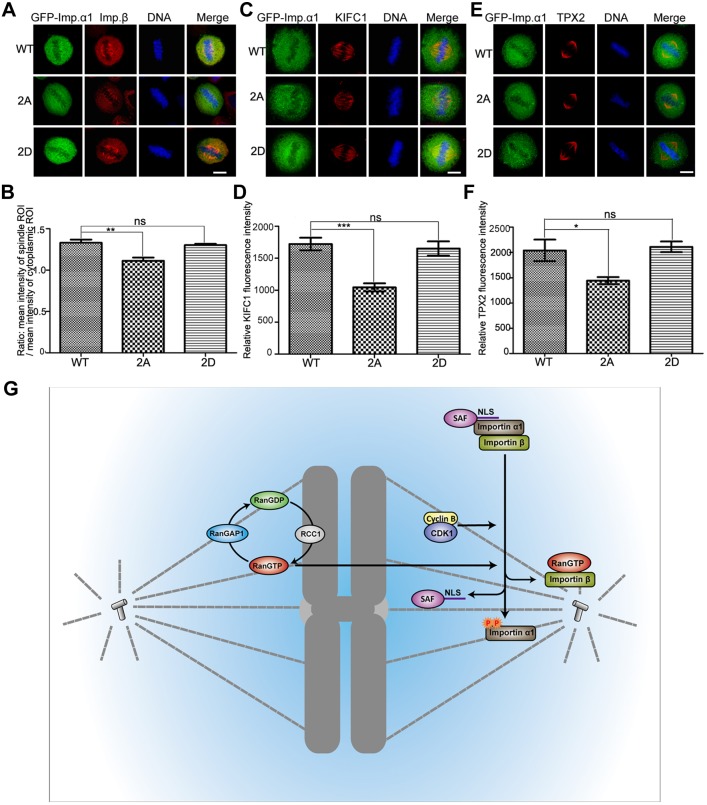
**Phosphorylation of importin-α1 is necessary for the localization of importin-β and SAFs on the mitotic spindle.** (A–F) HeLa cells were transfected with GFP–importin-α1 WT, GFP–importin-α1 2A and GFP–importin-α1 2D for 48 h and then were fixed with 3.7% PFA in PEM buffer and immunostained with anti-importin-β antibody (A) or were fixed with methanol and immunostained with (C) anti-KIFC1 or (E) anti-TPX2 antibody. DNA (blue) was stained using DAPI. Typical images are shown. Scale bars: 10 µm. (B) The ratio between the intensity of importin-β in spindle ROI and cytoplasm ROI was measured and statistically calculated. Experiments were repeated three times, and at least 100 cells were measured for each group. Results are mean±s.d. The intensity of spindle-located (D) KIFC1 and (F) TPX2 was measured by Volocity software (Perkin Elmer) and statistics calculated by GraphPad Prism5 software. *P<0.05, **P<0.01, ***P<0.001; ns, not significant (Student's *t*-test). (G) A model for the dual control of importin-regulated mitotic spindle assembly by Ran-GTP and CDK1–cyclinB1. The chromatin-localized guanine-nucleotide exchange factor RCC1 generates RanGTP at chromosomes, providing a spatial signal for spindle assembly. In addition, phosphorylation of importin-α1 by CDK1–cyclin B1 attenuates the interaction of importin-α1 with importin-β, promoting the release of NLS-containing spindle assembly factors (SAFs) from importin-α1.

Because the phosphorylation of importin-α1 promotes its release from importin-β, it was of interest to find out the effect of the phosphorylation of importin-α1 on the localization of importin-β, which is targeted to the spindle during mitosis (Ciciarello et al., 2004). By comparing the ratio of the mean intensity of the mitotic spindle and cytoplasmic regions of interest (ROIs), we found that the amount of importin-β localized on the mitotic spindle was significantly reduced in cells expressing GFP–importin-α1 2A, but not in cells expressing GFP–importin-α1 and GFP–importin-α1 2D (Fig. 8A,B). These results suggest that importin-β was detained in the cytoplasm by unphosphorylated importin-α1. Furthermore, the proper localization and function of SAFs to the mitotic spindle is also likely to require the release of these SAFs from the importin-α1–importin-β complex through the phosphorylation of importin-α1. To test this, GFP–importin-α1, GFP–importin-α1 2A and GFP–importin-α1 2D were expressed in cells, followed by immunofluorescence labeling for the SAFs. We observed that the amount of the NLS-containing SAFs KIFC1 and TPX2 on the mitotic spindle was significantly reduced in cells expressing GFP–importin-α1 2A, but not in cells expressing GFP-importin-α1 and GFP-importin-α1 2D (Fig. 8C–F). We also checked the spindle localization of both RANBP2 (Gilistro et al., 2017) and the importin α-independent spindle assembly factor HURP (Silljé et al., 2006) in GFP–importin-α1-expressing cells. We found that expression of the non-phosphorylation-mimicking 2A mutant reduced the spindle localization of RANBP2 but not HURP in comparison to the expression of WT GFP–importin-α1 and phosphorylation-mimicking mutants T9E and S62E (Fig. S6A,B). Thus, phosphorylation of importin-α1 appears to promote the recruitment of RanBP2 to the spindle but is not required for the importin-β-restricted targeting of HURP to the spindle. In summary, these results show that the phosphorylation of importin-α1 induces the release of NLS-containing SAFs to load onto the mitotic spindle, thereby promoting their function in mitotic spindle assembly.

## DISCUSSION

In eukaryotes with an open mitosis, the controlled boundary between the nucleoplasm and the cytoplasm breaks down at prometaphase, dispersing soluble nuclear proteins and exposing the chromosomes to cytoplasmic proteins. Importin-α1–importin-β dimers bind to NLS-containing SAFs during mitosis and this interaction restricts their functions. The spatial control of these interactions is critical for the proper formation of the mitotic apparatus during which MTs need to be specifically nucleated and stabilized only in the region of spindle formation, and proteins have to be recruited to the spindle for the regulation of chromosome segregation. The release of SAFs from inhibitory complexes with importin-α1–importin-β is directed by chromosomes through chromatin-generated Ran-GTP, which exists at a high concentration in the vicinity of chromosomes and a lower concentration farther away (Caudron et al., 2005; Clarke and Zhang, 2008; Kaláb et al., 2006). When the Ran-GTP gradient is disrupted, the spatial organization of spindle assembly is compromised (Caudron et al., 2005; Kaláb et al., 2006, 2002). The generation of Ran-GTP is linked to the progression of mitosis by the control of regulators, such as RCC1, through their phosphorylation by the major mitotic protein kinase, CDK1–cyclin B1 (Hutchins et al., 2004; Li and Zheng, 2004). However, additional mechanisms may provide temporal and spatial control of SAFs and thereby promote proper spindle assembly.

In this report, we have shown that the key partner and regulator of SAFs containing an NLS motif, importin-α1, is controlled during mitosis through phosphorylation at two sites by CDK1–cyclin B1, providing a general mechanism for the temporal and possibly spatial regulation of SAFs during mitosis. Activation of CDK1–cyclin B1 specifically during prometaphase/metaphase provides a temporal control, while concentration of CDK1–cyclin B1 to sites such as unattached kinetochores, chromatin, spindle MTs and centrosomes (Alfonso-Pérez et al., 2019; Bentley et al., 2007; Chen et al., 2008) could promote localized release of SAFs. Phosphorylation of importin-α1 by CDK1–cyclin B1 is likely to cooperate with dissociation of the NLS-SAF–importin-α1–importin-β complex by Ran-GTP but it also provides a mechanism for Ran- and chromatin-independent activation of SAFs (Fig. 8G).

We have shown that importin-α1 protein is highly expressed in mitosis, a finding that is consistent with cell cycle regulation of importin-α1 mRNA levels (Whitfield et al., 2002), whereas the amount of importin-β was maintained throughout the cell cycle. Although it may seem paradoxical that the level of an inhibitor of SAFs is increased in mitosis, distal MT nucleation and polymerization should be suppressed while the activity of SAFs is restricted to the area of spindle formation. Thus, the combination of a high level of importin-α1 in mitosis and its selective control by CDK1–cyclin B1 provides a mechanism to direct the spatial and temporal control of spindle assembly. Disruption of the equilibrium in functional importin-α1 levels through either its knockdown or, conversely, its overexpression as a non-phosphorylated form that cannot be controlled, disrupts proper spindle formation. It is also interesting that the protein level of importin-α1 is reduced in S phase, although the functional consequences are as yet unclear. Other importin α isoforms might meet the requirements for the nuclear import of proteins, such as DNA replication and repair factors during this phase of the cell cycle, whereas importin α1 has a relatively more significant role during mitosis.

Mitotic phosphorylation of importin-α1 occurs at two sites in the human protein, T9 and S62. T9 is only conserved in humans, but there is a potential serine phosphorylation site at this position in some other mammals. S62, together with a proline residue at the +1 position as required for recognition by CDK1–cyclin B1, is conserved from fruit fly to humans, suggesting that the mitotic phosphorylation of importin-α1 plays a conserved regulatory role in animals (Fig. S3F). Importin-α1 consists of a flexible N-terminal IBB domain (in which T9 is located) containing an auto-inhibitory motif that regulates the binding of importin-α1 with an NLS, and an elongated domain comprising 10 Arm repeats containing major and minor NLS-binding sites. The two domains are connected by a presumably disordered loop in which S62 is located**.** The efficient interaction of importin-β and NLS-containing substrates via these two domains is important for importin-α1 to act as a regulator for NLS cargoes (Kobe, 1999). Based on our results, both the phosphorylation in the IBB domain and in the disordered loop connecting the IBB domain and NLS-binding domain can alter the binding affinity with importin-β and NLS cargoes. In the absence of importin-β, the auto-inhibitory motif in the IBB domain binds to the NLS-binding region due to the flexibility of the loop connecting these two domains, further reducing the binding with NLS cargoes (Kobe, 1999). If the flexibility of the connecting loop between the IBB and the NLS-binding domain is altered by phosphorylation of S62, the subsequent altered intramolecular interaction between IBB and NLS-binding domain might change the ability to bind importin-β. Further structural investigations may be of interest to accurately describe why the alteration affects the interaction of importin-α1 with importin-β and NLS cargoes.

We have also shown that importin-α3, an isoform with restricted NLS-containing partners, is not regulated like importin-α1 by phosphorylation by CDK1–cyclin B1. One significant partner of importin-α3 during mitosis is RCC1, and this interaction is controlled instead through phosphorylation by CDK1–cyclin B1 of the NLS motif of RCC1 (Li and Zheng, 2004; Moore et al., 2002; Sankhala et al., 2017). Thus, CDK1–cyclin B1 generally controls SAFs that interact with importin-α1 through phosphorylation of the importin, whereas the same kinase controls a key importin-α3 interaction specifically through phosphorylation of the partner protein. Interactions between importin-α3 and other non-phosphorylated cargoes could therefore be maintained during mitosis. Interestingly, the interaction between importin-α1 and a mitotic partner, TPX2, which is disrupted by phosphorylation of importin-α1, appears to be unusual compared to that exhibited by most NLS cargoes, since it is binds primarily to the minor NLS-binding site on importin-α1 (Giesecke and Stewart, 2010). Thus, it is possible that phosphorylation of importin-α1 preferentially releases SAFs that interact with importin-α1 in this way while maintaining interactions with other cargoes that are not required for spindle assembly and which might interfere with the process. Dephosphorylation of importin-α1 towards the end of mitosis might be necessary for efficient nuclear envelope formation (Lu et al., 2012).

To summarize, our results reveal that both the expression and phosphorylation of importin-α1 by CDK1–cyclin B1 are required for spindle assembly in mitosis, and this mechanism provides a level of spatial and temporal control of this critical process. Normally, this mechanism would enhance the proper function of the mitotic spindle and chromosome segregation during cell division. However, defects in this mechanism in proliferating cells could result in loss of fidelity in chromosome segregation and thereby promote the generation of chromosome instability, which is associated with cancer and other genetic diseases.

## MATERIALS AND METHODS

### Cloning, expression and purification

The cDNA of importin-α1 was cloned into pEGFP-C2 (Clontech), pET-28a (Novagen) and pcDNA3.1-Myc (Invitrogen) to generate GFP-, His-, and Myc-tagged importin-α1. The pEGFP-C2-importin-α1 and pcDNA3.1-Myc-importin-α1 plasmids were used as templates for site-directed mutagenesis to generate the mutations at T9A, S62A, T9E, S62E, 2A and 2D. The cDNA of importin-β was cloned into pGEX4T-1 (Novagen) to generate GST-tagged importin-β. pET28a-KIFC1 887-end was kindly provided by Shuli Zhang and pEGFP-NT was a kind gift from Qinying Liu (both at College of Life Sciences of Peking University, China). *E. coli* expressing His–importin-α1 and His–KIFC1 887-end were purified by TALON Metal Affinity Resin (BD Biosciences Clontech) according to the manufacturer's instructions. *E. coli* expressing GST–importin-β was purified using glutathione–Sepharose-4B (Pharmacia, Pfizer Inc., NY) according to the manufacturer's instructions. The proteins were desalted using an Amicon ultral-4 centrifugal filter (Millipore) and were resuspended in PBS buffer (137 mM NaCl, 2.7 mM KCl, 10 mM Na_2_HPO_4_ and 2 mM KH_2_PO_4_).

### Antibodies and inhibitors

The following antibodies were used for immunofluorescence (IF) and western blotting (WB): rat monoclonal anti-importin-α1 (1:100 for IF, 1:1000 for WB; 2G7; ab22534; Abcam), rat polyclonal anti-importin-α1 (1:100 for IF, 1:1000 for WB; I9658; Sigma-Aldrich), mouse monoclonal anti-importin-α (1:100 for IF, 1:1000 for WB; I1784; Sigma-Aldrich), mouse monoclonal anti-importin-β (1:100 for IF, 1:1000 for WB; 3E9; ab2811; Abcam), rabbit polyclonal anti-importin-β (raised against GST-tagged full-length importin-β and used at 1:1000 for WB), anti-histone H3 phospho-S10 (1:2000 for WB; 05-1336; Sigma-Aldrich), anti-α-tubulin (1:100 for IF, 1:2000 for WB; T9026; Sigma-Aldrich), anti-GFP (1:2000 for WB; sc-9996; Santa Cruz Biotechnology), anti-KIFC1 (in-house polyclonal, raised against his-tagged 887-end of KIFC1 and used at 1:100 for IF, 1:1000 for WB), rabbit polyclonal anti-TPX2 (1:100 for IF, 1:1000 for WB; 12245; Cell Signaling Technology), anti-NuMA (1:1000 for WB; ab109262; Abcam), anti-Ran (1:5000 for WB; 610340; BD Biosciences) and anti-Myc (1:1000 for WB; MABE282; Sigma-Aldrich). Kinase inhibitors were used at the following concentrations: 10 µM purvalanol A(Sigma-Aldrich), 9 µM Ro-3306 (Sigma-Aldrich), 0.1 µM BI2536 (Axon Medchem), 2 µM ZM447439(Selleck), 0.5 μM LY294009 (Selleck).

### Cell culture, transfection and synchronization

Authenticated HeLa cells were obtained from the ATCC and were maintained in DMEM supplemented with 10% fetal bovine serum, and were cultured in an incubator at 37°C in the presence of 5% CO_2_. Cells were tested routinely to ensure they were free from Mycoplasma. Transient transfections were carried out in cells using Lipofectamine 2000 (Invitrogen) with the indicated constructs. For G1/S arrest, the cells were synchronized by performing a double-thymidine blockade. Briefly, cells were incubated with 2 mM thymidine (Sigma-Aldrich) for 16 h, followed by washing and releasing cells into fresh medium for 9 h, and then incubating with 2 mM thymidine for another 16 h. The cells were then released into normal medium and harvested at different time points or into medium containing 100 ng/ml nocodazole for 12 h to harvest the cells in mitosis. The cells in mitosis were then released into normal medium and harvested at different time points. For monastrol arrest, double-thymidine blocked cells were released into fresh medium for 9h, then treated with 50 μM monastrol (Sigma-Aldrich) for 3 h.

### Phosphorylation analysis by Phos-tag™ SDS-PAGE

Phos-tag™ is a phosphate-binding reagent that is used for the separation, purification and detection of phosphorylated proteins (Kinoshita et al., 2006). We used this product (Phos-tag AAL-107, Wako Pure Chemical Industries) to detect phosphorylated proteins in SDS-PAGE gels. The samples were loaded into the wells of 8% SDS-PAGE with Mn^2+^-Phos-tag™ in a resolving gel. After electrophoresis, the gel was soaked in transfer buffer with 1 mM EDTA for 10 min and then in transfer buffer without EDTA for another 10 min before transfer onto a PVDF membrane using a wet-tank method followed by western blotting.

### Immunoprecipitation

To determine the interaction between proteins, immunoprecipitation was carried out from cell lysates using immunoprecipitation buffer (0.5% NP-40, 20 mM Tris-HCl pH 7.4, 500 mM NaCl, 0.5 mM EGTA, 10 mM NaF, 2 mM NaVO_3_, 10 mM β-glycerophosphate, 10 µg/ml leupeptin, 0.7 µg/ml pepstatin A, 1.5 µg/ml aprotinin and 1 mM PMSF). For GFP-tagged precipitations, 5 µg of rabbit anti-GFP antibody (raised in the laboratory in rabbit using His-tagged GFP) was incubated with 15 µl of prewashed Protein-A–Sepharose beads (GE Healthcare) at 4°C for 1 h. The beads were then washed three times with immunoprecipitation buffer. Mitotic HeLa cells transfected with GFP-tagged plasmids were lysed on ice for 30 min in immunoprecipitation buffer. The lysates were then centrifuged at 13,200 rpm for 15 min to collect the supernatants to be incubated with beads at 4°C for 1 h. The beads were then washed five times with immunoprecipitation buffer and then were analyzed by western blotting. For endogenous protein precipitations, the indicated antibodies and non-transfected HeLa cells were used. The experiments were carried out as stated above.

### Preparation of cell extracts

HeLa cells were grown in 15 cm cell culture plates. Asynchronous cells and mitotic cells were collected. After being washed in pre-cooled EBS buffer (80 mM β-glycerophosphate, pH 7.2, 20 mM EGTA, 15 mM MgCl_2_, 100 mM sucrose, 1 mM DTT, and 1 mM PMSF), the cells were resuspended in an equal volume of pre-cooled EBS buffer and incubated in an ice bath for 10 min. The resuspended cell pellets were sonicated in an ice bath and then were centrifuged at 16,560 ***g*** for 30 min at 4°C. The supernatants were collected and transferred into new pre-cooled 1.5 ml centrifuge tubes and were centrifuged again at 16,560 ***g*** for 15 min at 4°C. The asynchronous and mitotic supernatants were collected and transferred into new pre-cooled 1.5 ml centrifuge tubes. The concentrations of these extracts were measured with a spectrophotometer and then were stored in liquid nitrogen.

### *In vitro* transcription and translation of importin-α1

Importin-α1 DNA was subcloned into pcDNA3.1-Myc (which contains a T7 promoter) and then was transcribed and translated in rabbit TNT reticulocyte lysate using a TNT Quick Coupled Transcription/Translation Kit (Promega). Next, 500 ng of the pcDNA-importin-α1 WT or mutant DNA was mixed with 20 µl of TNT lysate and 1 µl of [^35^S]-labeled methionine (1000 Ci/mmol, Amersham) and then was made up to 25 µl with dH_2_O, followed by incubation at 30°C for 90 min. The *in vitro*-translated (IVT) proteins were then used immediately or stored at −20°C for later use.

### Phosphorylation of IVT proteins by cell extracts and purified recombinant kinase

To determine the kinase activity in HeLa cell extracts that could phosphorylate recombinant importin-α1 proteins, 2 μl of radiolabeled IVT protein was incubated with 20 µg of cell extracts in a 10-µl kinase reaction containing 50 mM Tris-HCl pH 7.5, 100 µM DTT, 100 µM ATP and 100 µM MgCl_2_. The reactions were incubated at 30°C for 30 min. The reactions were quenched by the addition of 30 μl of 2× SDS sample buffer (50 mM Tris-HCl pH 6.8, 2% SDS, 10% glycerol in dH_2_O, 2.5% Bromophenol Blue and 10% β-mercaptoethanol were added prior to use), boiled for 3 min and spun for 5 min at 13,520 ***g*** (Eppendorf 5434 centrifuge; rotor HL 077). The samples were separated by Phos-tag™ SDS-PAGE and conventional SDS-PAGE. Following SDS-PAGE, the gels were dried on a gel dryer (VWR International) and were subjected to autoradiography. For phosphorylation by CDK1–cyclin B1, 20 µg of kinase (Upstate) and 2 µl of [^35^S]-labeled IVT protein were incubated at 30°C for 30 min in buffer containing 50 mM Tris-HCl pH 7.5, 100 µM DTT, 100 µM ATP and 100 µM MgCl_2_. The reactions were terminated by the addition of 2× sample buffer and equimolar MnCl_2_. The samples were boiled at 100°C for 3 min, followed by centrifugation for 5 min at 15,870 ***g***. The samples were subjected to Phos-tag™ SDS-PAGE and conventional SDS-PAGE. The gels were then dried on a gel dryer for 2 h. [^35^S]-labeled proteins were detected by autoradiography of the dried gel after overnight exposure at −80°C.

### Autoradiography

For radiolabeled proteins separated by SDS-PAGE, the gels were dried onto 3-mm chromatography paper at 80°C for 2 h under vacuum. The gels were exposed to X-omat film (Kodak) overnight at −80°C or longer. Radiating bands were viewed after the films were developed.

### RNA interference

To knockdown importin-α1 *in vivo*, chemically synthesized siRNAs were used. The siRNA sequence were as follows: negative control siRNA, 5′-UUCUCCGAACGUGUCACGU-3′; importin-α1 siRNA 1#, 5′-CCAAGAUUAU**U**C**U**GGUUAU-3′ (U→C mutations for siRNA resistance are indicated in bold). Where stated, anti-Luc (luciferase) siRNA (Dharmacon) was also used as a negative control.

For KPNA2 siRNA-resistant plasmids, we generated point mutations in GFP–importin-α1 using site-directed mutagenesis as stated above. Next, 100 pmol of siRNA and 1 μg of resistant plasmids were transfected into HeLa cells using 5 μl of Lipofectamine 2000 (Invitrogen) for 72 h. The cells were then collected for western blotting or immunofluorescence and microscopy.

### Immunofluorescence and microscopy quantification

HeLa cells grown on coverslips were washed with PBS buffer and then were fixed with precooled methanol for 6 min on ice. After rehydration in TBS and blocking with 3% BSA for 30 min, the cells were incubated with primary antibodies overnight at 4°C. Next, the cells were washed with TBS three times and were incubated with secondary antibodies for 1 h at room temperature. Finally, after washing with TBS, the cells were stained with 1 μg/ml of DAPI and were observed under a DeltaVision microscopy imaging system. The spindle length and fluorescence intensity were measured by Volocity software (Perkin Elmer). Statistical quantifications were performed using GraphPad Prism5 software.

### Time-lapse live-cell imaging

HeLa cells were grown in glass-bottomed dishes and were transfected with the indicated siRNA for 72 h or plasmids for 24 h. The cells were then placed in a DeltaVision live cell station for automated time-lapse imaging. Images were captured every 3 min under a fluorescence microscope. The average time from nuclear envelope breakdown (NEBD) to sister chromosome separation of cells was calculated and statistically analyzed using GraphPad Prism5 software.

## Supplementary Material

Supplementary information
