# Supplementary material for: Phosphorylation of importin-α1 by CDK1–cyclin B1 controls mitotic spindle assembly
Source: J Cell Sci. 2019 Sep 23;132(18):jcs232314. doi: 10.1242/jcs.232314 (PMC6765185; doi:10.1242/jcs.232314)
Supplement: Supplementary information [file joces-132-232314-s1.pdf]

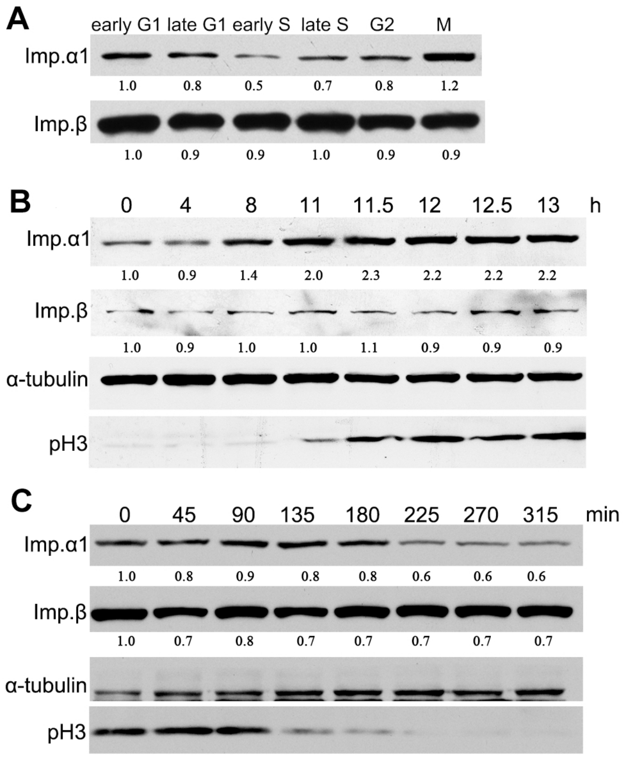

Figure S1. Cell cycle-dependent expression of importin-α1 protein.

(**A**) The expression level of importin-α1 protein is regulated in a cell cycle-dependent manner. Early S, late S and G2 phase cell extracts were prepared from synchronous HeLa cells at 1 hour, 4 hours and 8 hours after release from double-thymidine blockade; M phase, early G1 and late G1 cell extracts were prepared from synchronized HeLa cells at 0 hours, 4 hours and 9 hours after release from nocodazole arrest. Cell extracts were subjected to SDS-PAGE and were analyzed by immunoblotting with antibodies as indicated. (**B**) The endogenous importin-α1 protein level dramatically increased in cell lysates after entry into mitosis. HeLa cells were arrested at the G1/S using double thymidine blockade, followed by release at different time points (hours). Cell extracts were subjected to SDS-PAGE and then were analyzed by immunoblotting with antibodies as indicated. (**C**) The endogenous importin-α1 protein level was decreased in cell lysates after exit from mitosis. HeLa cells were arrested at mitosis using double-thymidine blockade, followed by release into nocodazole blockade, and then release at different time points. pH3 (Histone H3 phospho-S10) was detected to indicate mitosis. Cell extracts were subjected to SDS-PAGE and then were analyzed by immunoblotting with antibodies as indicated. The numbers under the bands refer to the relative grey value intensity.

Journal of Cell Science • Supplementary information

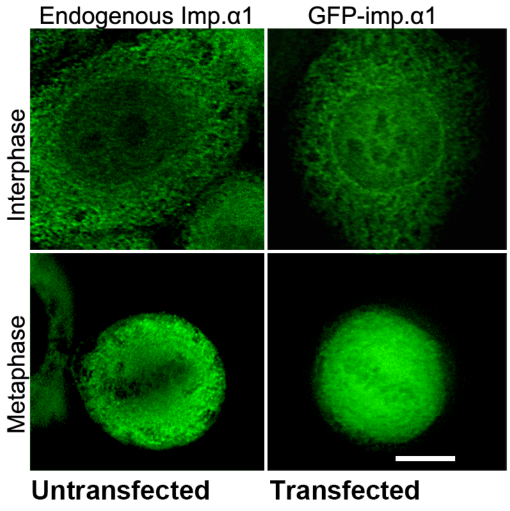

Figure S2. Localization of importin-α1 in interphase and metaphase cells.

HeLa cells grown on cover slips in cell culture dishes were transfected with GFP-importin-α1 plasmids for 24 hours. Untransfected HeLa cells were fixed with pre-cooled methanol and immunostained for endogenous importin-α1. Transfected HeLa cells were fixed with pre-cooled methanol. Cells were then observed under a DeltaVision microscopy imaging system. Scale bar, 10 μM.

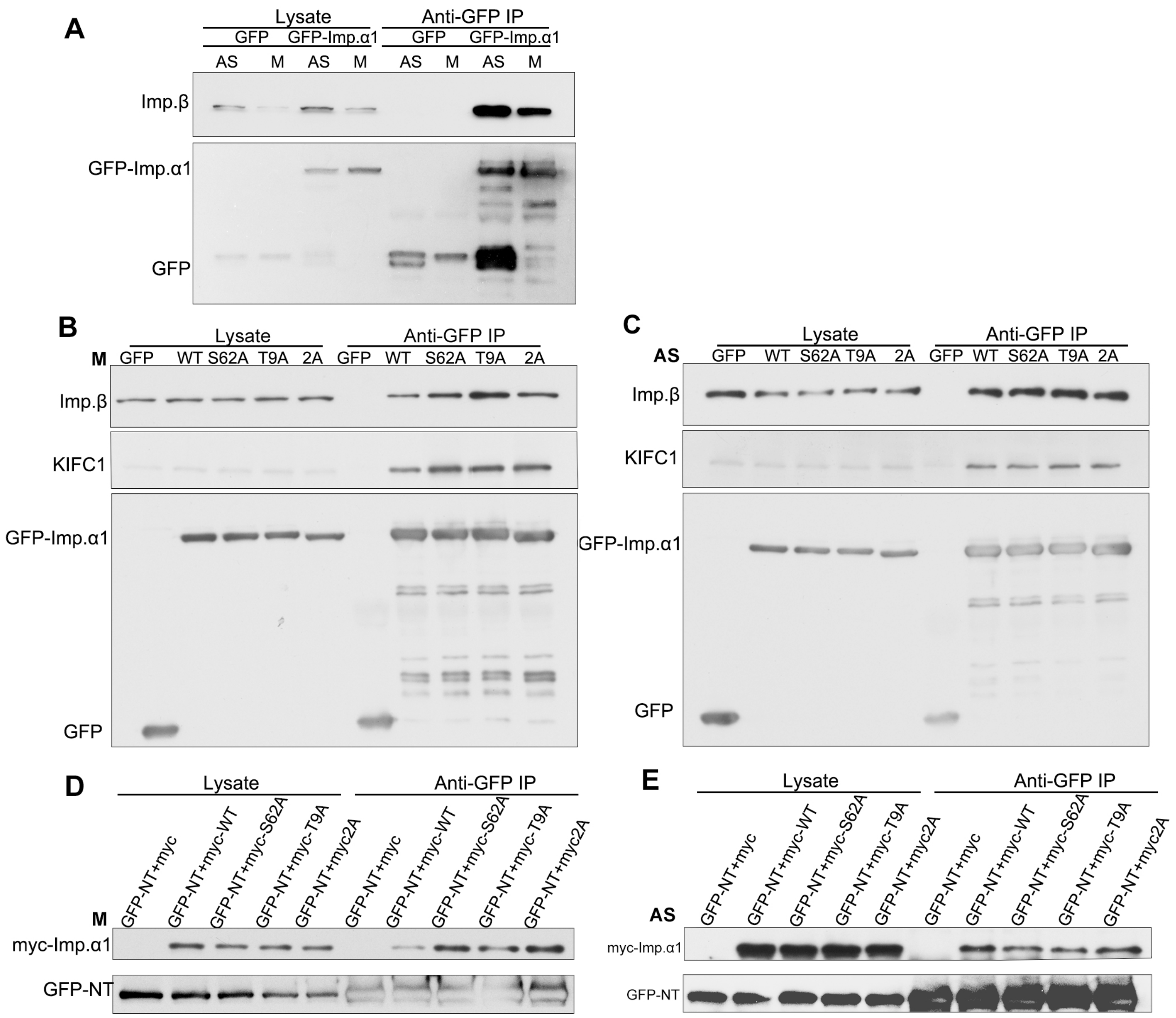

Figure S3. Interaction of importin-α1 with importin-β and NLS-containing SAFs.
(**A**) HeLa cells were transfected with GFP or GFP-importin-α1. Asynchronous or nocodazole-arrested mitotic cells were prepared for anti-GFP immunoprecipitation and were subjected to SDS-PAGE and immunoblotting for importin-β and importin-α1. Cell lysates used for precipitations are shown in the left panels. (**B**) HeLa cells were transiently transfected with either GFP, GFP-importin-α1 WT, GFP- importin-α1 S62A, GFP-importin-α1 T9A or GFP-importin-α1 2A and then were arrested in mitosis by 100 ng/ml of nocodazole for 17 hours. GFP-importin-α1 was immunoprecipitated from mitotic cells using an anti-GFP antibody, followed by immunoblotting for importin-β, KIFC1, TPX2, NuMA and GFP. Cell lysates used for the precipitations are shown in the left panels. (**C**) HeLa cells were transiently transfected with either GFP, GFP-importin-α1 WT, GFP-importin-α1 S62A, GFP-importin-α1 T9A or GFP-importin-α1 2A. GFP-importin-α1 was immunoprecipitated from asynchronous cells using an anti-GFP antibody, followed by immunoblotting for importin-β, KIFC1, TPX2, NuMA and GFP. Cell lysates used for the precipitations are shown in the left panels. (**D**) HeLa cells were co-transfected with NLS-containing fragment GFP-NT and myc-tagged importin-α1 or mutants (myc-importin-α1 WT (myc-WT), myc-importin-α1 S62A (myc-S62A), myc-importin-α1 T9A (myc-T9A) and double mutant myc-importin-α1 2A (myc-2A)) and then were arrested in mitosis by 100 ng/ml of nocodazole for 17 hours. GFP-NT was immunoprecipitated from mitotic cells using an anti-GFP antibody, followed by immunoblotted for myc and GFP. (**E**) HeLa cells were co-transfected with the NLS-containing fragment GFP-NT and myc-tagged importin-α1 or mutants (myc-importin-α1 WT (myc-WT), myc-importin-α1 S62A (myc-S62A), myc-importin-α1 T9A (myc-T9A) and double mutant myc-importin-α1 2A (myc-2A)) and then were collected as asynchronous cells. GFP-NT was immunoprecipitated from asynchronous cells using anti-GFP antibody, followed by immunoblotting for myc and GFP.

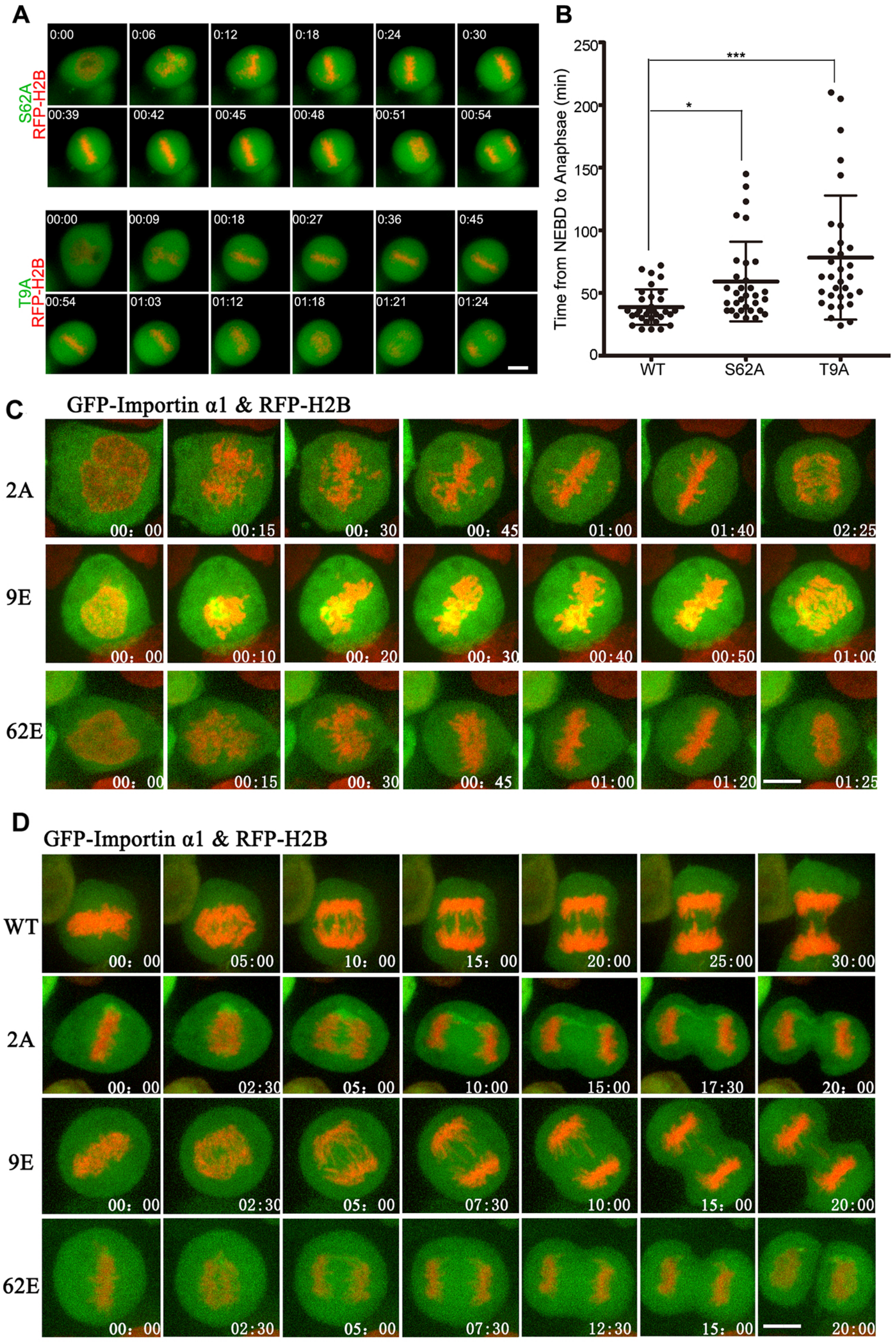

Figure S4. Phosphorylation of importin-α1 at Thr9 and Ser 62 is required for mitotic progression.

(**A**) HeLa cells were co-transfected with GFP-importin-α1 S62A or T9A and RFP-H2B (as a chromatin marker) and then were subjected to automated time-lapse live-cell fluorescence imaging. The GFP signals indicate cells transfected with GFP-importin-α1. The red signals indicate H2B. Scale bar, 10 μM. (**B**) Statistics showed the average time from NEBD to anaphase. N=50 cells per group. ***P < 0.001, *P < 0.05. Error bars, standard deviations. **(C and D)** HeLa cells were cultured on glass-bottom dishes, transfected with RFP-H2B and GFP-importin α1 WT, 2A, 9E or 62E, and treated with thymidine for 17 hours to block these cells at S phase. These cells were then released into fresh medium for 9 hours and analysed by live imaging. Images were taken every 2.5 minutes. Note that the time between NEBD and chromosome congression was prolonged in 2A- but not in 9E- and 62E- expressing cells (C). Once cells started anaphase mitosis was exited within 30 minutes, regardless of whether they were expressing 2A, 9E, or 62E mutants (D). Scale bar, 10 μM.

Journal of Cell Science • Supplementary information

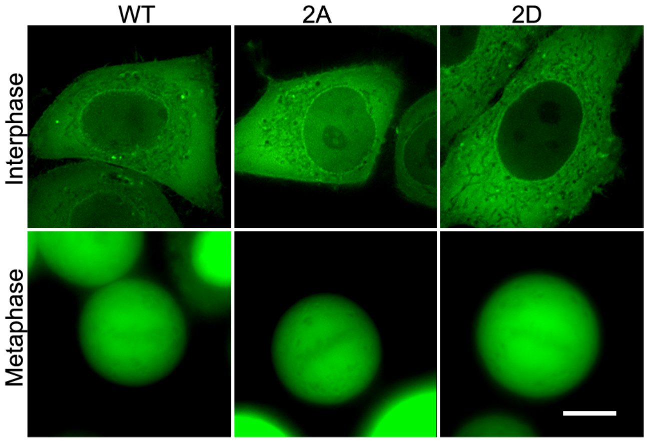

Figure S5. Localization of GFP-importin-α1 and mutants in live cells.

HeLa cells were co-transfected with GFP-importin-α1 WT, GFP-importin-α1 2A or GFP-importin-α1 2D and then were subjected to live-cell fluorescence imaging. Typical interphase cells and round mitotic cells are shown. Scale bar, 10 μM.

Journal of Cell Science • Supplementary information

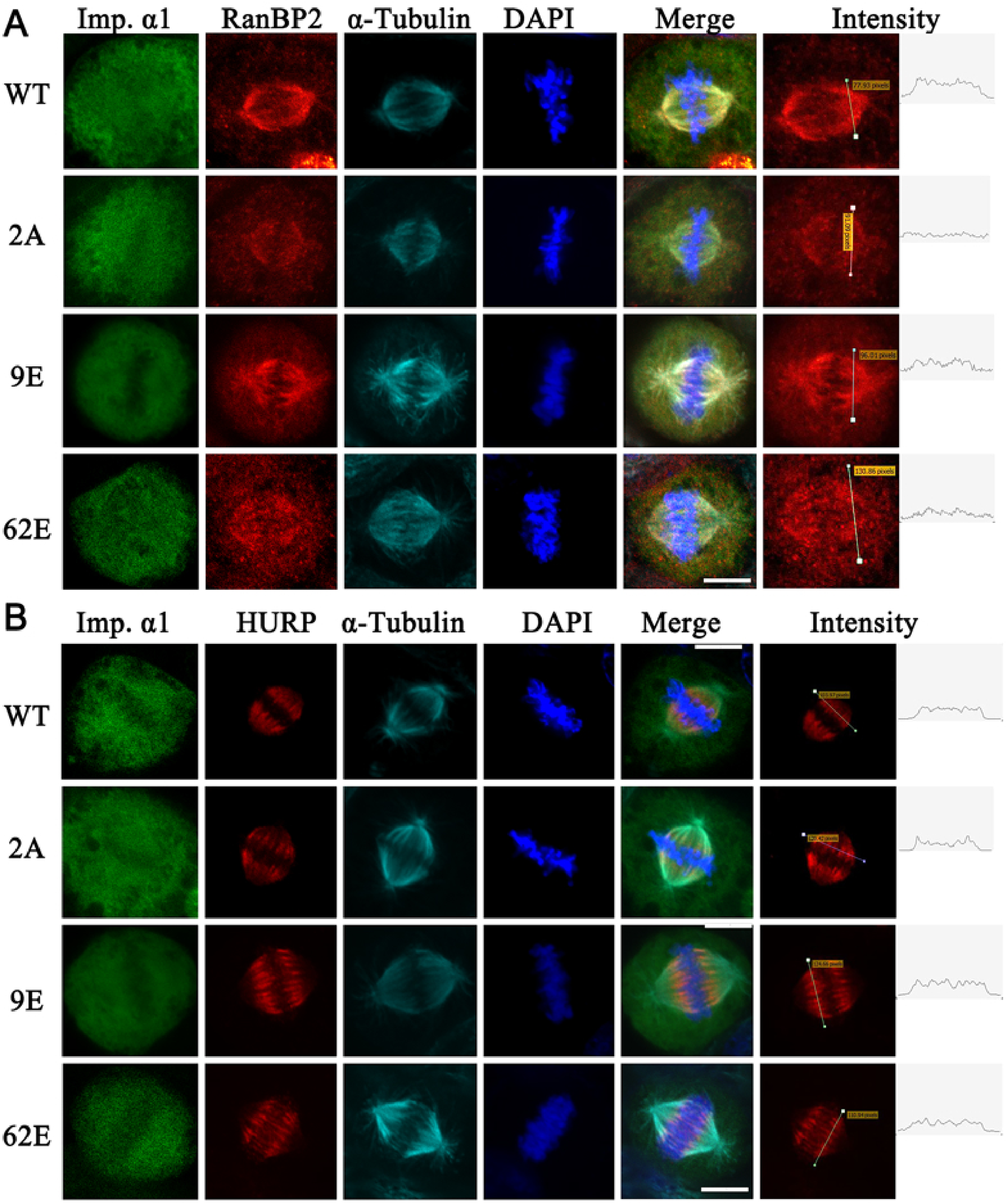

Figure S6. Phosphorylation of importin-α1 is required for localisation of RanBP2 but not HURP to the mitotic spindle.

HeLa cells were transfected with GFP–importin α1 WT or 2A plasmid. 24 hours after transfection, the cells were fixed with methanol and immunostained with anti- α-tubulin, RanBP2 and HURP antibodies. The relative fluorescence intensity of RanBP2 and HURP on a cross section of the spindles, indicated by the straight line on the images, was plotted, respectively. The relative fluorescence intensity was measured using Volocity software (Perkin Elmer). Note that the localisation of RanBP2 in 2A- but not WT- expressing cell was reduced; and in contrast, the localisation of HURP on the spindles was not affected by 2A or WT expression. Scale bar, 10 μM.
